# Patients’ perspectives on urethral bulk injection therapy and mid-urethral sling surgery for stress urinary incontinence

**DOI:** 10.1007/s00192-018-3644-0

**Published:** 2018-04-19

**Authors:** Fenne M. Casteleijn, Sandra E. Zwolsman, Claudia R. Kowalik, Jan-Paul P. W. R. Roovers

**Affiliations:** Department of Obstetrics and Gynecology, Academic Medical Center, University of Amsterdam, Meibergdreef 9, 1105 AZ Amsterdam, The Netherlands

**Keywords:** Stress urinary incontinence, Urethral injection therapy, Mid-urethral sling surgery, Treatment preference

## Abstract

**Introduction and hypothesis:**

The aim of this study was to identify all treatment decision factors that determined the preference for peri-urethral bulk injection therapy (PBI) or mid-urethral sling (MUS) surgery in patients with primary stress urinary incontinence (SUI). Second, we explored what patients expect from treatment for SUI and whether patients would consider PBI as a primary treatment option.

**Methods:**

In a qualitative design, 20 semi-structured, face-to-face interviews were conducted in women with primary SUI. Exclusion criteria were: previous PBI or MUS surgery; predominating urgency. Interviews were guided by three open-ended questions and a topic list. PBI treatment and MUS surgery were described in detail, and the efficacy was stated as 70% and 90%, respectively. Data saturation was reached when no new treatment decision factors were identified in three consecutive interviews. Interviews were audiotaped and fully transcribed. Thematic analysis by a coding process was done independently by two researchers.

**Results:**

Sixteen procedural, personal, professional, social and external treatment decision factors were identified. Regarding expectations about treatment for SUI, women believed ‘becoming dry’ was wishful thinking. The majority of patients accepted a small degree of persistent urinary incontinence after treatment. Regardless of their treatment preference, patients indicated that women should be informed about PBI as a primary treatment option.

**Conclusion:**

Patients with primary SUI are open to PBI as an alternative treatment option even with lower cure rates compared with MUS surgery performed under general or spinal anesthesia. Patients indicated that women with primary SUI seeking treatment should be informed about PBI as a treatment option.

## Introduction

Stress urinary incontinence (SUI), defined as the involuntary leakage of urine on exertion or sneezing or coughing, is a major public health issue affecting up to 45% of women [1–3]. Several treatment options for SUI are available, and treatment decisions are based on international guidelines and both the physician and patient preference. Pelvic floor muscle training (PFMT) is considered a valuable first option, since PFMT has a negligible risk of complications and achieves a patient-reported cure rate of 56% [4]. Mid-urethral sling surgery is considered the first surgical option because of the high efficacy rates [5–7]. Besides PFMT and MUS surgery, various alternative treatment options are available, including peri-urethral injection therapy (PBI). However, even though patients with SUI want to be informed about their treatment options and to be involved in treatment decision-making [8, 9], they are often unaware of PBI as a treatment option.

The hypothesis of the efficacy of PBI is that it compresses the urethra and improves urethral coaptation by injecting a synthetic biomaterial peri-urethrally. One benefit of PBI is that the procedure can be performed under local analgesia in an office setting. Second, the Cochrane Review reports that bulk injection therapy has a better safety profile compared with open surgery [10]. Although a prospective cohort study of PBI after 1-year follow-up showed promising results with cure rates of 70% [11], randomized trials comparing PBI and MUS surgery and long-term follow-up data are lacking. Therefore, PBI is not recommended as a first-line therapy and is mainly offered to patients who have a contraindication for MUS surgery or to patients with complex or recurrent SUI [10, 12–14]. Petrou et al. showed, however, that injection therapy could still be the first choice treatment for patients who attach more value to a less invasive procedure [15]. This suggests that the cure rate is not always decisive in selecting the right treatment for the right patient.

To explore whether patients consider PBI a reasonable primary treatment option for SUI, one should first understand the patients’ perspectives or the expectations that underlie their motivation for PBI instead of standard treatment. This insight increases the understanding of patient decision-making and helps physicians to address the correct items in shared decision-making.

In this qualitative study, we primarily aimed to identify all treatment decision factors that determine the preference for PBI and MUS surgery in patients with primary SUI. Second, we aimed to explore what patients expect from treatment for SUI in general and whether patients would consider PBI as a primary treatment option for SUI.

## Materials and methods

This qualitative study focused on patient perspectives on factors to take into account when choosing between PBI and MUS surgery. The methods and results of this study are reported according to the consolidated criteria for reporting qualitative research (COREQ) [16].

### Recruitment

Patients with SUI were recruited at a tertiary urogynecologic center in The Netherlands where about 700 women with urinary incontinence are seen per year. To be eligible, women had to be Dutch-speaking and seeking treatment for SUI. Patients with predominant urgency incontinence or a history of MUS surgery or PBI treatment were not eligible. It was hypothesized that patients of different ages and different perceived severities of symptoms would have different perspectives concerning SUI treatment. Therefore, the investigator selected the participants until wide ranges of ages and of patients with mild, moderate and severe SUIs were adequately represented. This method of recruitment is called purposive sampling [17]. Eligible patients were informed about the study by an information leaflet, and those not willing to participate were asked to give a reason. The sample size was completed when data saturation occurred, meaning that more interviews would not lead to more information [18, 19]. Data saturation was reached when no new treatment decision factors were observed in three consecutive interviews [20]. The ethics board confirmed that the Dutch ‘Medical Research Involved Human Subjects Act’ did not apply to this study and that no further review was required.

### Topic list and interview

The interviews had a face-to-face format, and the interviewer relied on a semistructured interview guide with three open-ended questions and a framework of topics to discuss. This open format allowed following the narrative of patients and picking up on all factors they brought up rather than following fixed or loose sequences of predefined questions, as in structured or semi-structured interviews, respectively [21]. Predetermined topics were: anesthesia, efficacy, complications, safety, setting, recovery and postoperative pain, re-interventions and sexual function. The contents of the topic list were compiled by an expert panel of two urogynecologists (CK; JR) and an experienced researcher in the field of qualitative research (SZ). The interviews were conducted by a female researcher with a medical doctor’s degree (FC) who pilot tested the topic list on two women with SUIs. After pilot testing, no new topics emerged, and therefore no revisions were made to the topic list. The interviews took approximately 60 min and took place at the patient’s home to ensure a safe environment. Prior to the interview, written informed consent of the patient was obtained, and the patients’ characteristics were collected. The global impression of severity (PGI-S), a validated one-item questionnaire with a four-point Likert scale from normal to severe, was used to assess the subjective severity of symptoms [17].

The interview started by exploring patients’ expectations about treatment in general by using the first open-ended question, *“What do you expect from a treatment for SUI?”* Then, the interviewer informed participants about the procedure and complications of MUS surgery and PBI, as shown in the Appendix. A non-degradable polydimethylsiloxane bulking agent (Urolastic®; Urogyn BV Nijmegen, The Netherlands) used at the institute was described as the PBI treatment. Patients were not yet informed about the efficacy of the procedure to specifically perceive the patients' perceptions of the procedure and safety of the treatment. Using the second open-ended question, *“Which factors would you take into account if you could choose between PBI and MUS surgery?”*, the decision-making factors were explored when choosing between PBI and MUS surgery. The topic list was modified when new factors emerged. After the decision factors had been explored, the participants were informed about the efficacy of the treatment: 70% and 90% for PBI and MUS surgery, respectively [7, 11]. The efficacy was defined as subjective cure: no symptoms of urine leakage during laughing, sneezing, coughing and physical exercise. It was mentioned that the long-term efficacy of PBI was unknown. The interview evaluated how the difference in efficacy influenced the women’s treatment preference. In addition, patients' perspectives on MUS surgery in a daycare setting performed under local analgesia with combination sedation were explored. Third, the women’s opinions about PBI as a primary treatment option were explored: *“Would you consider PBI a primary treatment option?”*

At the end of the interview, the interviewer gave a summary of the interview, which the participant could correct or complete.

### Data analysis

All interviews were audiotaped and transcribed verbatim by the interviewer. The data were analyzed by two researchers who worked independently [FC; ZS] with the help of the MaxQdA12 software package. Deductive content analysis was used for pre-determined decision factors, and inductive content analysis was used to identify additional decision factors from the remaining narratives [22]. Thematic analysis was done as follows [23, 24]: (1) Interviews were read line by line and the decision factors were marked (open coding) [25]. (2) The relationship of the codes was identified by categories and subcategories by means of constant comparison (axial coding) [26, 27]. (3) The categories were combined with an iterative process and domains developed (selective coding) [26]. The participants received feedback on the study findings.

## Results

From November 2015 until July 2016, 33 women were approached for participation, and 11 women declined. Two patients were excluded from the study by purposive sampling because the patients had minimal complaints of SUI and this group of patients was already overexposed. The major reason for refusing participation was private matters; one woman indicated that she could not express herself properly. After interviewing 20 women, no new treatment decision factors were observed in three consecutive interviews, meaning data saturation had been reached and therefore no new women were approached for participation. The patient characteristics in Table 1 show the variety in age, cultural and educational background, duration of incontinence symptoms and subjective severity of incontinence symptoms (PGI-S).

**Table 1:** Patient characteristics

Characteristic	*N* = 20
Age in years *median (range)*	49 (23–88)
Duration of symptoms in months *median (range)*	60 (6–964)
Use of anti-incontinence material *n (%)*	15 (75)
Previous therapy for SUI *n (%)*	
None	1 (5)
PFMT	18 (90)
Unknown	1 (5)
Sandvik Severity Scale* *n (%)*	
Mild	0
Moderate	10 (50)
Severe	10 (50)
PGI-S** *n (%)*	
Normal	0
Mild	11 (55)
Moderate	5 (25)
Severe	4 (20)
Ethnicity *n (%)*	
Dutch	17 (85)
Chinese	1 (5)
Colombian	1 (5)
Belgian	1 (5)
Education *n (%)*	
Primary school	1 (5)
Secondary school	14 (70)
University	5 (25)
Marital status *n (%)*	
Married	13 (65)
Living together	2 (10)
Single	1 (5)
Widow	4 (20)
Profession *n (%)*	
Full time	2 (10)
Part time	9 (45)
Unemployed	3 (15)
Retired	6 (30)
Parity *median (range)*	2 (0–4)
Premenopausal *n (%)*	11 (65)
Sexual active *n (%)*	12 (60)

*The Sandvik Severity Scale is a validated index that scores the severity of urinary incontinence by multiplying the outcome points of two questions regarding the frequency and amount of urinary loss

**PGI-S: patients' global impression of improvement is a validated scale to assess their ‘subjective severity of urinary tract conditions'

### Treatment decision factors

**Fig. 1:**
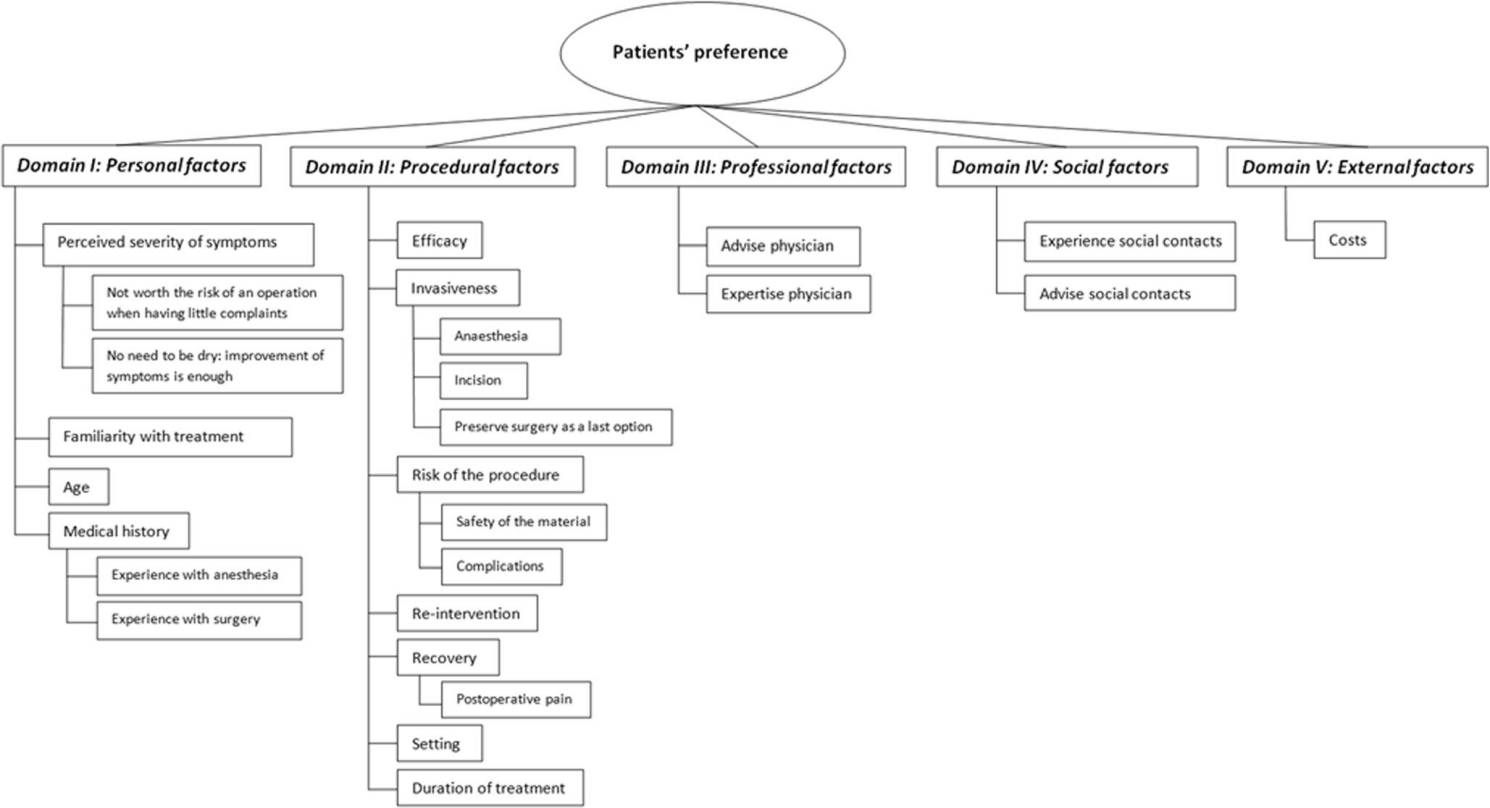
Code tree of domains and treatment decision factors

Sixteen treatment decision factors, categorized in five domains, determined the patients’ treatment preference between PBI and MUS surgery (Fig. 1). Predetermined treatment decision factors from the topic list were all categorized in domain ‘procedural factors.’ Sexual function was deleted as a treatment decision factor because this was a factor related to undergoing treatment in general and did not discriminate between PBI and MUS surgery. After data analysis, ten new treatment decision factors and four new domains were identified. The top three most mentioned decision factors of the largest domains are described in the text. Table 2 shows illustrative quotations of reasons to opt for PBI or MUS surgery or to be indecisive.

**Table 2:** Selected illustrative quotations

Treatment preference	Treatment decision factor	Quotation
Preference for PBI therapy	Having few complaints	*“I think I would not even consider the tape (refers to MUS surgery) because I think it still does not outweigh the problems I have at this moment…the operation may be more efficacious, but that does not pursuade me to undergo surgery”* (P10, 40 years)
	No need to be dry	*“I am not like: I should be dry until the last drop. That is not a goal for me…So, then I still prefer the bulk injection, because when you have improvement* (refers to symptoms of SUI) *it can be acceptable for everyday life”* (P5, 41 years)
	Reserve surgery as a last option	*“If it does not work* (refers to PBI)*, you can still choose the operation”* (P9, 47 years)
	Safety of the material	*“I would not dare* (refers to MUS surgery)*, because you quickly hit something… and then those incisions…with all that scar tissue…just the idea that this tape can never be removed…So I would go for the bulk injection”* (P16, 34 years)
	Avoid anesthesia	“*If I had to choose now, I would choose the bulk injection, but that is due to the fact that the operation is through an epidural or general anesthesia”* (P7, 23 years)
	Avoid incision	*“I would still prefer the bulk injection, purely because they do not have to cut; I’m always afraid they will hit things"* (P9, 47 years)
	Less invasive	*“The bulk injection is more convenient. And less heavy. There are people who go to clinics to fill up certain things, well…this reminds me a bit of that”* (P1, 50 years) *“Although it is less effective, I would still try this first* (refers to PBI) *because it is less invasive”* (P4, 37 years)
Preference for MUS surgery	Familiarity with treatment	*“I think the bulk injections are scary…I do not know, maybe because you have heard little about it”* (P13, 71 years)
	Efficacy	*“The most important thing for me is it must be efficacious”* (P20, 48 years)
	Safety of the material	*“Sounds more chemical and scarier* (refers to PBI)*…imagine it’s leaking or so…what can happen? What are the risks? Can it move? Could it be that your body does not accept it? That it will be expelled itself? Can it get into your bladder? That kind of uncertainties”* (P4, 37 years)
	Avoid local analgesia	*“I think I would go for the operation, because then I am not awake* (refers to general anesthesia)*”* (P18, 86 years)
	Re-intervention	*“If the chance is 15% that you have to still must undergo anesthesia anyhow* (refers to excision of PBI)*, perhaps I would say, I will do the operation immediately”* (P14, 32 years)
	Expertise physician	*“I think I would choose the operation, because it is more efficacious and there is more experience with the operation. But I would talk to my husband about it”* (P6, 55 years)
Indecisive	Age	*“They do not have any experience with this* (refers to PBI) *in older women, so I think that is a risk to take. The injections seem painful. With an operation you do not feel anything, that is beneficial. But then afterwards…*(refers to recovery)*. And one women I know has been operated on twice, but is still as incontinent as before, so it did not help. Why should I take that risk at my age?”* (P17, 88 years)
	Advice from physician	*“I do not know…I will wait for what the doctor tells me, I will wait for their advice”* (P2, 82 years)

This table shows patient quotations that reflect their reasons for preferring PBI treatment or MUS surgery or being indecisive

#### Domain I: personal factors

The patients’ perceived severity of symptoms influenced the treatment preference. Women opted for PBI even if it would be less effective if they considered their symptoms not severe enough to undergo surgery or when they intended to achieve a reduction of symptoms rather can complete cure of incontinence.

Especially older patients mentioned age as a reason to prefer PBI over MUS surgery because they intended to avoid general or spinal anesthesia. On the other hand, also older women expressed fear of silicon-induced complications.

Finally, the familiarity of the treatment was a major factor influencing patient preference. MUS surgery was considered a well-known procedure, but PBI treatment was unfamiliar to the patients. Lack of confidence about PBI treatment tended to arise from unfamiliarity and was therefore a decision factor for opting for MUS surgery.

#### Domain II: procedural factors

The minimally invasive characteristic was a repeated decision factor for choosing PBI. Although surgery had a higher success rate, some patients were keen to try the least invasive procedure first and reserve surgery as the last option. When further exploring the term ‘invasiveness,’ patients valued ‘incision’ and ‘anesthesia’ as the most incriminating factors. An incision was considered a risk factor for infection, bleeding and extensive fibrosis and was often dominant in patients’ trade-off of treatment decision factors. The preference for type of anesthesia was very personal and based on previous experiences or fear of complications. Although local analgesia was generally perceived as appealing and a reason to choose PBI, one woman preferred MUS surgery because of previous painful experiences with local analgesia. When MUS surgery was offered as a procedure under local analgesia with sedation, most women perceived this to be a preferred setting, but only one woman who preferred PBI switched her preference to MUS surgery. Women who did consider local analgesia not beneficial had different reasons for this. Either women were too anxious about the pain during the procedure or anxious about being awake during the procedure, or they still considered the sedation a disadvantage. Finally, for some women the type of anesthesia was just not important in their treatment decision-making. The risk of the procedure and especially the safety of the material influenced the patient treatment preference. With respect to PBI, women worried about ‘injecting something’ because the substance could be resorbed, migrate or cause a foreign body reaction. Many questions and thoughts arose concering the safety of the PBI material: *“it sounds chemical and more scary.” “Can it leak like silicone breasts?” “Can it be carcinogenic?”* With respect to MUS surgery, women worried about fibrosis, infection, persistent pain or the inability to remove the whole sling.

Finally, the efficacy was a treatment decision factor. Table 3 reflects the treatment preference in relation to patients' age and severity of symptoms before and after informing them about the efficacy. One patient switched her preference from PBI to MUS surgery after being informed about the difference in efficacy. Two patients first preferred PBI, but became indecisive after receiving information on efficacy. Two patients were indecisive, but afterwards preferred MUS surgery. Six patients still preferred PBI therapy, although they knew it was less effective. Seven patients preferred MUS surgery before and after informing them about the efficacy rates.

**Table 3:** Hypothetical treatment preference related to the efficacy

Treatment preference	Before information on efficacy	After information on efficacy	PGI-S*^I^	Age^I^ *median (range)*
PBI *n (%)*	10 (50)	6 (30)	Mild: 5 Moderate: 1	38 (23–47)
MUS surgery *n (%)*	7 (35)	11 (55)	Mild: 5 Moderate: 3 Severe: 3	55 (24–86)
Indecisive *n (%)*	3 (15)	3 (15)	Mild: 1 Moderate: 1 Severe: 1	82 (50–88)

This table shows the number and percentage of patients that preferred PBI treatment or MUS surgery or were indecisive before and after informing them about the efficacy of PBI treatment and MUS surgery

*PGI-S: patients' global impression of improvement is a validated scale to assess their subjective severity of urinary tract conditions

I: PGI-S and age presented in the table reflect the population of patients after receiving information on efficacy

Patients found the outpatient setting, less postoperative pain and quicker recovery of PBI beneficial, but these factors were less dominant in treatment decision making. Also, when MUS surgery was presented as day-care ambulant treatment, this was found appealing, but was not decisive in their treatment decision-making.

#### Domain III: professional factors

The advice and expertise of the physician were taken into account when choosing between PBI and MUS surgery. Especially older and indecisive women attached great value to advice from physicians. Women assumed that physicians were more experienced performing the MUS procedure than PBI and therefore expected a better outcome from MUS surgery.

#### Domain IV: social factors

Especially experiences from other patients, but also advice from social contacts and family contributed to the patients’ preference.

#### Domain V: external factors

One woman enquired about the reimbursement and possible costs of the treatments.

### General expectation concerning treatment

Regarding SUI treatment expectations, women believed ‘becoming dry’ was wishful thinking. As long as the remaining incontinence did not involve more than drops requiring one pad a day, or using small pads instead of large ones, they were satisfied. They accepted the consequences of giving birth and increasing age and did not expect that treatment could completely cure their incontinence symptoms. Other women expected to achieve more personal goals such as “playing field hockey with the children.” A minority of the women said that they would not accept any urine loss after treatment.

### Perspective on PBI as a primary treatment option

Regardless of the patients' treatment preferences, the lower efficacy of PBI treatment did not prevent them from believing that PBI should be offered as a primary treatment option. Women indicated that physicians should inform women about all possible treatment options, including PBI treatment, so they can carefully weigh the benefits and disadvantages of both treatments and make a well-informed decision. One woman indicated that, if she had more influence in decision-making, she would be more confident during her treatment. Another woman added that the physician’s advice was a must.

## Discussion

This study shows that patients with primary SUI consider PBI a valuable alternative treatment option even though it has lower cure rates compared with MUS surgery performed under general or spinal anesthesia. Second, patients indicated that PBI should be incorporated in shared decision-making and offered to all women with SUI.

In the counseling process for SUI treatment, attention mainly focuses on procedural factors such as the chance of cure, type of anesthesia, setting and recovery. However, this study shows that patients also take into account personal, professional, social and external factors when making a treatment decision for PBI or MUS surgery. Regardless of the chance of cure, the patients’ preference for PBI or MUS surgery was strongly based on aversions to or concerns about the treatment method (respectively injection or incision) or the safety of the used material (respectively silicon or mesh). For example, some patients just disliked the idea of injections. On the other hand, safety issues regarding mesh was a decision factor for choosing PBI.

A major decision factor for choosing PBI was its minimally invasive character. Although MUS surgery is generally known as a minimally invasive procedure, some patients preferred PBI because they considered general or spinal anesthesia or the incision for MUS surgery too invasive. PBI treatment was found an appealing intermediate option between conservative management and MUS surgery. Therefore, some patients wanted to reserve the most invasive procedure (MUS surgery) as the last treatment option, a phenomenon that was also described in a qualitative study by Milne et al. comparing conservative treatment versus surgery [28]. Therefore, patients do not always prefer the treatment with the highest cure rate. This is supported by Petrou et al. who showed that patients prefer injectable therapy over tension-free vaginal tape surgery with a mean success rate as low as 34% [15].

Major decision factors involved in patients preferring MUS surgery were: the higher chance of cure, a one-session procedure, the familiarity with the treatment and safety concerns about PBI treatment. A qualitative study on patients’ treatment preferences in women with pelvic floor disorders also reported that women with SUI want to have the treatment with the highest chance of long-term success, even if it is more invasive [29].

The patients’ general expectations of treatment were the hope of achieving improvement of their symptoms, and only a few expected a complete cure. Moreover, women indicated having specific treatment goals, such as ‘playing field hockey with my children again.’ This is in line with other studies showing that treatment goals for patients with urinary incontinence are very personal and subjective [30–34]. Therefore, even if PBI cure rates were significantly lower cure than for mid-urethral sling procedures, it can not be concluded that PBI would not meet patients' treatment goals.

In this qualitative design, there are several uncertainties concerning the generalizability of the results. First, the results are not applicable for women who have recurrent SUIs after MUS surgery, as we excluded those women from this study. We purposely chose to include treatment-naive women to prevent influences of previous experiences on their perception. Second, the success rates and re-intervention rates mentioned by the interviewer are hypothetical and could be different from daily practice counseling. With respect to PBI, we used a 15% chance for both the re-injection rate and excision rate based on outcomes from clinical studies of a non-degradable polydimethylsiloxane bulking agent [11, 35]. However, these re-intervention rates can differ significantly among different bulking agents. For example, the re-injection rate of the urethral bulking agent polyacrylamide hydrogel (PAHG) can be up to 35% [36]. Since re-intervention was a decision factor for choosing MUS surgery, the differences in re-injection percentages of the different bulking agents could have influenced the women’s preferences. Third, although data saturation for decision factors occurred, the sample size was small considering the wide range of patients with SUI. So, it might be that some patient characteristics have been underexposed, despite the fact that purposive sampling was used. For example, not all ethnicities were represented, and cultural factors may not have been identified. This effect might be minimal since a systematic review showed similar management strategies for urinary incontinence among different racial groups. However, PBI was not evaluated in this systematic review [31]. Fourth, we did not share details about the time-dependent characteristics of the efficacy of both interventions. Finally, an interview is a snapshot of women’s perspectives, and their perspectives may change over time.

A strength of the study, by using a qualitative design, is that not only subtle distinctions of interpretations can be made, but also the broad spectrum of the patients’ perspectives is highlighted. To structure the patient perspectives, domains were used to categorize the treatment decision factors. The layout of categorization (personal, procedural, professional, social and external domains) is reported in other studies [37, 38].

PBI treatment was introduced as a promising alternative treatment option for SUI. However, because of safety issues, high re-injection rates and the lack of durable results, it is not widely accepted as a valuable treatment option. Although a systematic review including 26 cohort studies of two currently used bulk materials shows subjective success rates ranging from 66 to 89.7% and objective success rates ranging from 25.4–73.3% at 12-month follow-up, randomized controlled trials with MUS surgery are missing [39]. As a consequence of the lack of evidence, the precise indication for PBI is still unclear. This study shows that patients would consider PBI a primary option when a cure rate of 70% after 1 year is achieved. This outcome is an argument for comparable studies to determine whether current bulk materials meet this level of success. One meta-analysis that compared PBI with open surgery showed significantly inferior results for PBI regarding objective cure; however, subjective outcomes were not significantly different [14]. Future studies therefore should include both subjective and objective outcomes.

This study shows that patients have different reasons to consider PBI as a primary treatment option compared with MUS surgery. In addition, patients indicated that PBI should be offered to all women with SUI. Comparable studies are however needed to objectify whether current bulking agents do meet cure rates as used in this study and to determine the precise indication for PBI treatment. Since the patient has gained a participant role when it comes to healthcare decisions, one should still identify the patient's perspective when tailoring treatment for SUI. The treatment decision factors identified in our study will help physicians to address the correct items in the discussion with the patient about the treatment of choice.

## Appendix. Treatment description of PBI and MUS surgery

The procedure and complications of PBI and MUS surgery were explained in detail by the interviewer using an information leaflet, but withholding information about the efficacy of the treatment options. MUS surgery was described as an operation that required general or spinal anesthesia and hospital admission for 1 or 2 days. Postoperative analgesia was usually necessary. The recovery period was described as 1 week. Postoperative lifestyle advice (such as no lifting, no cycling and no physical exercise) was applicable for 4–6 weeks. Possible complications involved: urinary retention, urgency incontinence, urinary tract infection, hemorrhage during or after surgery, wound infection, persistent pain, dyspareunia and exposure of the sling through the vaginal wall. The PBI that was presented was a non-degradable polydimethylsiloxane bulking agent. PBI was described as a procedure under local analgesia injecting a non-absorbable silicon substance at four locations around the urethra. The procedure was performed in an outpatient setting, and hospital admission was not necessary. The procedure time was set at 20 min. Postoperative analgesia was seldom indicated, and the recovery was 1 to 2 days. Postoperative lifestyle changes as described in MUS surgery were also applicable to women treated with a PBI. The risk of having to undergo a re-injection of two extra silicon deposits in case of recurrent SUI was set at 15%. Possible complications involved: urinary retention, urgency incontinence, urinary tract infection, wound infection, exposure or expulsion of the bulking agent, persistent pain and dyspareunia. The risk of having to remove one or more silicon deposits because of the aforementioned complications was set at 15%. Excision of one or more deposits could indicate spinal or general anesthesia and hospital admission.
